# A Patient Journey Map to Improve the Home Isolation Experience of Persons With Mild COVID-19: Design Research for Service Touchpoints of Artificial Intelligence in eHealth

**DOI:** 10.2196/23238

**Published:** 2021-04-12

**Authors:** Qian He, Fei Du, Lianne W L Simonse

**Affiliations:** 1 Department of Design Organisation & Strategy Faculty of Industrial Design Engineering Delft University of Technology Delft Netherlands

**Keywords:** COVID-19, design, eHealth, artificial intelligence, service design, patient journey map, user-centered design, digital service solutions in health, home isolation, AI, touchpoint

## Abstract

**Background:**

In the context of the COVID-19 outbreak, 80% of the persons who are infected have mild symptoms and are required to self-recover at home. They have a strong demand for remote health care that, despite the great potential of artificial intelligence (AI), is not met by the current services of eHealth. Understanding the real needs of these persons is lacking.

**Objective:**

The aim of this paper is to contribute a fine-grained understanding of the home isolation experience of persons with mild COVID-19 symptoms to enhance AI in eHealth services.

**Methods:**

A design research method with a qualitative approach was used to map the patient journey. Data on the home isolation experiences of persons with mild COVID-19 symptoms was collected from the top-viewed personal video stories on YouTube and their comment threads. For the analysis, this data was transcribed, coded, and mapped into the patient journey map.

**Results:**

The key findings on the home isolation experience of persons with mild COVID-19 symptoms concerned (1) an awareness period before testing positive, (2) less typical and more personal symptoms, (3) a negative mood experience curve, (5) inadequate home health care service support for patients, and (6) benefits and drawbacks of social media support.

**Conclusions:**

The design of the patient journey map and underlying insights on the home isolation experience of persons with mild COVID-19 symptoms serves health and information technology professionals in more effectively applying AI technology into eHealth services, for which three main service concepts are proposed: (1) trustworthy public health information to relieve stress, (2) personal COVID-19 health monitoring, and (3) community support.

## Introduction

### COVID-19

In December 2019, a new type of coronavirus causing acute respiratory syndrome (COVID-19) was discovered in Wuhan. COVID-19 spread rapidly around the world and was designated as a Public Health Emergency of International Concern by the World Health Organization (WHO) on January 30, 2020 [1]. By 2 AM CEST (Central European Summer Time) on June 29, 2020, there had been 9,962,193 confirmed cases and 498,723 confirmed deaths [2]. Although lockdown measures have been eased in many countries, a WHO COVID-19 situation report (June 28) still shows a rising trend [3]. This worldwide pandemic has had a far greater impact than expected, and the upward trend is likely to continue in the near future until effective vaccines and antivirals are introduced.

### Home Isolation for 80% of Persons With Mild COVID-19 Symptoms

Looking back at the early months of the outbreak, the rapid spread of COVID-19 and the growing number of patients placed a burden on unprepared medical systems worldwide [4,5]. Some countries such as China and Spain set up mobile cabin hospitals to relieve the pressure on their hospitals [6-8]. However, to ensure that the limited health care resources were spent on urgent cases involving severe symptoms, most countries decided that persons with mild symptoms could be isolated at home for a self-recovery trajectory [9]. According to the early WHO studies, about 80% of COVID-19 cases present mild symptoms, and most of these patients should typically be able to recover at home [10-13]. Therefore, disease control centers in various countries (eg, the United Kingdom, the United States, the Netherlands, Italy, and Canada) directed persons with mild COVID-19 symptoms to stay at home and contact their general practitioner by phone instead of directly visiting the hospital. New guidelines for home care were developed by the WHO and countries’ public health departments to present the proper measures for the home care of patients [13-17].

### Strong Demand for eHealth Services

As an alternative solution to conventional health care services, the uptake of eHealth services rapidly expanded during the COVID-19 pandemic [18]; the main fields of application have been telemedicine, remote patient monitoring, and triage and risk assessment [19]. Enabling better response to the pandemic, such digital health care solutions not only reduce the risk of disease transmission thanks to the provision of remote medical care services but also hold the promise to improve the mental health of isolated patients with distance guidance [18-20]. Initial studies have shown that, since the outbreak began, the frequency of internet searches related to “online medical care” has increased significantly, and the public’s interest in eHealth is rising as the number of infected cases climbs [21,22]. Although the limited capacity of the existing eHealth service systems cannot immediately meet this growing demand [22], there is no doubt that the COVID-19 pandemic will impact the current service provision of medical institutions and lead to an accelerated transition to digital health care.

### Potential of Artificial Intelligence

The large number of digital health applications that have been released in response to the COVID-19 outbreak includes a growing number of artificial intelligence (AI) tools; these include tools that make use of natural language data processing and machine learning with big data lakes, such as in decision support agents, advanced self-diagnoses tooling, and AI-enabled mental health interventions [23-27]. These AI tools have the potential to add new service options to remote health care modes such as remote assessment, remote diagnosis, remote interaction, and remote monitoring [24]. The main touchpoints of AI technology appeared to be mobile phone apps, wearable devices, and chat robots [25]. Prior research in the context of public eHealth and disease prevention has found that AI technology improves patients’ health conditions more efficiently [24]. Moreover, application of AI appeared to enable more personalized care pathways based on personal health profiles [18]. The COVID-19 pandemic requires existing health care models to have better integration, delivery, and distribution capabilities, and thus, new requirements for AI in eHealth have been put forward [18,28,29]. Thus far, the main fields that AI technology has been applied to during the pandemic is early detection and diagnosis of infections, personal contact tracking, case and mortality prediction, drug and vaccine development, reducing the workload of medical staff, and other aspects of controlling and managing the spread of the virus [23]. Overall, the emerging AI tools have the potential to perform a useful role in combating COVID-19, and in particular, AI has the potential to improve the quality of home care by providing more personalized, sophisticated, and continuous medical services [18,24].

### A Lack of Understanding of the Real Needs: Home Isolation Experiences

Despite the potential of AI in digital health services, many attempts to integrate AI technology into health have failed [24,30]. The primary reason for this is that the development of AI tools and applications is predominantly focused on technical and functional aspects, and largely ignores the demands of users and contextual aspects [31]. Data scientists and health professionals usually start developing AI technology without exploring the patient perspective in advance, that is, the health experience and needs of the users. Often users are only involved in providing feedback after a system solution test is carried out or after the final digital service has been released [30]. However, without sufficient understanding of the user experience, AI service applications will not be capable of solving the real and serious problem of a lack of useful value [32,33]. In other words, the real needs of patients with mild symptoms of COVID-19 have not yet been identified. Illustrative of this lack of understanding the real needs, most of the current machine learning algorithms behind decision support tools are too opaque and difficult for users to reconstruct [34]. When AI machines provide treatment suggestions, the mysteriousness of this process basically causes users, including health professionals and patients, to question the result [35]. If AI technology is expected to be used to improve existing eHealth service capabilities, the key actors should focus more on the users rather than technology to reduce the gap between technology and user, and to improve the usefulness of AI [24]. Corresponding findings related to cancer conditions indicated that AI technologies provide a way to transition from a traditional aperiodic “snapshot” monitoring approach to a continuous and longitudinal monitoring paradigm, increase patients’ engagement in their care, and facilitate doctor-patient interaction pathways [36]. In particular, a study that applied machine learning and natural language processing techniques on social media data from online cancer support groups provided new insights toward informed decision making on personalized health care delivery [37]. Likewise, an increasing amount of AI demonstrators evidence a new service potential of AI applications that are hardly used yet in enhanced service providing. To be able to meet personal patients’ needs, in-depth research is required.

### Design for Better Supported Home Isolation Experiences

To overcome the barrier of the lack of knowledge on what is useful and what is not, AI research and development should involve user-centered design methods to gain insights into the real experiences and needs of users [38], as well as apply a person-centered perspective to construct an explainable AI and make the AI process more transparent and comprehensible to multiple users, including patients and health professionals [39]. As Xu [32] stated, “a useful AI is defined as an AI solution that can provide the functions required to satisfy target users' needs in the valid usage scenarios of their work and life,” which means the user experience should be deeply understood before the AI development starts. However, most of the current research is mainly focused on persons with severe COVID-19 or patients treated at hospitals. We found no literature that explicitly describes the home isolation experience of persons with mild COVID-19 symptoms. Thus, knowledge of this area is still required. This paper intends to address this gap by elaborating on the entire process of the home isolation experience of people with mild COVID-19 symptoms—from infection to recovery—and then extracting in-depth insights for AI concepts in eHealth. Specifically, our research question is how can we improve the home isolation experience of persons with mild COVID-19 symptoms through eHealth services with AI technology?

## Methods

### Design Research

We employed a design research method in which a qualitative approach was used to explore the home isolation experience of persons with mild COVID-19 symptoms because this is a relatively new field [40]. Our design research had a phenomenology perspective that rests on the philosophical assumptions of studying people’s experiences in their daily living, viewing these experiences as conscious [41]. This phenomenon study provides a real and comprehensive description of the home isolation experience of persons with mild COVID-19 symptoms, which is needed to obtain insights into their user needs and tasks [40], and to find touchpoint interaction needs for the useful application of AI in eHealth.

#### Patient Journey Mapping

Patient journey mapping is a method of design research for developing health care services from a patient perspective [42-45]. The purpose is to capture insights into a patient’s activities, interactions, feelings, and motivations throughout the personal health care journey, and to generate insights into user values and dilemmas that lead to the identification of real and underlying problems that must be solved through the successful application of innovative solutions [42,45]. The final journey map visualizes the commonly shared patient experiences and includes both physical, rational, and functional aspects of the patient experience as well as the emotional, interactional, and feelings aspect of patient experiences [42]. The design quality of the patient journey map is determined by its properties to visualize the knowledge and insights about the patient’s experience and enable sympathy of the viewers by placing them in the perspective of the patient [44].

Prior research exemplified concise journey maps of visually compelling stories, distilling research into all aspects of personal experience and informing the reflections on the steps and approach laid out in the patient journey method [42]. The design of the patient journey map in this study contributed new knowledge on the home isolation experience of persons with mild COVID-19 symptoms and thereby provided the view of the patient and enabled a deep understanding of their whole experience from the onset of illness to recovery [45]. The patient journey map depicts all steps of the journey to gain a better understanding of the whole journey, taking the arising needs of patients into account, and uncovers new research fields for relevant AI applications [46].

### Data Collection

Data on the home isolation experiences of persons with mild COVID-19 symptoms was collected from the top viewed personal video stories on YouTube and their comment threads. As researchers, the global pandemic meant that we were bound by the necessity to engage in social distancing and limit interpersonal contacts. Therefore, we chose to use personal video stories instead of interview techniques. YouTube, one of the major online video sharing platforms, has become recognized as a valuable social media source for personal stories about health and disease [47]. We chose YouTube as the data source because, compared to other social media platforms such as Twitter, Instagram, and Tik Tok, its content richness and the completeness of the stories presented on it enables more detailed data collection about an entire journey experience. Videos also allowed us as researchers to better understand the feelings of the patients from their nonverbal movements and expressions [48]. The personal video stories and comment threads provided us with a new research opportunity to investigate actively shared experiences instead of relying on actively obtained experiences from interviews. From a researcher’s perspective, YouTube videos eliminated research bias and brought to light unexpected information that those posting them consider important from their personal perspective. The difficulties involved in the use of YouTube concerned the analysis of different narrative structures that posed challenges in extracting, coding, and synthesizing commonly shared experiences.

#### Sample Strategy

Purposive sampling was selected for the in-depth study of the experiences of persons with mild symptoms who were in home isolation [49]. As most YouTubers are young people, who account for a large proportion of persons with mild symptoms, they do not represent the total population of persons with mild COVID-19 symptoms across socioeconomic classes and ethnic and cultural groups [47]. We were likely to find personal video stories on home isolation experiences from the young YouTubers population representation because they are more likely to become the first embracer of new technology-based services [50]. The YouTubers we selected for the study after using the search terms “COVID-19,” “experience/personal stories,” and “home isolation” were, first, persons who shared their COVID-19 health conditions over a period of consecutive days or weeks*.* Second, we looked for influential videos with more than 100,000 views. Third, we selected stories that were perceived to be authentic and did not have any negative comment about their authenticity from more than 100,000 views, and we excluded those videos that did. In addition, we checked if the content stayed available (dated December 16, 2020) after the YouTube COVID-19 Policy and Security. Fourth, to achieve data saturation, we selected 5 as a suitable sample size to cover the wide range of possible experiences [51]. To some extent, this study is representative. In particular, the use of the YouTube platform comes with constraints for validity, as it can only represent the internet population and, in our sample of the videos with the most viewers, those who use the English language and live in the region of the Americas and Europe [47,48]. Further constraints relate to the fact that upper middle class Americans of European decent are more likely to post [47]. Table 1 lists the characteristics of the sampled personal video stories. To dig further into how online social support influences persons with mild COVID-19, we also collected and analyzed the top 50 popular comments on each of the videos.

**Table 1:** Sample of personal video stories and comment threads.^a^

No.	YouTuber’s age (years)	Mild COVID-19 health and well-being condition (when uploaded)	Language of personal video story	YouTuber’s region	Upload date in 2020	Video length (MM:SS)	Views, n	Likes, n	Dislikes, n	Comments, n
1	20-30	Sick	English	Americas	March 10	11:48	661,846	58,000	935	7082
2	20-30	Almost recovered	English	Americas	March 25	10:03 + 13:37	395,069 + 61,749	6677 + 1629	567 + 50	2350 + 725
3	20-30	Almost recovered	English	Europe	April 11	43:15	248,659	5637	333	1208
4	20-30	Almost recovered	English	Americas	April 9	11:27	183,711	3527	254	1315
5	41	Sick	English	Europe	April 5	10:50	246,814	15,000	263	3315

^a^Data collected from YouTube on May 26, 2020 (checked on December 16).

#### Ethics

This study was reviewed by the Human Research Ethics Committee of the Delft University of Technology [52]. In our sample strategy, we did not involve vulnerable groups of children or patients older than 65 years. As the personal video stories are published on the YouTube platform, we considered that they, in principle, constitute a publicly available data source for research [53]. For further confirmation, we emailed all 5 YouTubers to obtain consent and received 2 replies with affirmative answers. To minimize potential harm, we kept their identity anonymous and did not describe their characteristics and contexts in detail.

### Data Analysis

In the data analysis, triangulation was used by clustering the data from the observation and the transcripts of videos and the comment threads [54].

#### Patient Journey Mapping

To fully understand the experience of these patients during home isolation, both generic and personalized experiences were analyzed based on the similarities and differences between the patients, respectively [42]. The analysis of the indicated *stage duration* was visually mapped to make the similarities and differences between personal journeys transparent. The *symptoms* of each patient at different stages were analyzed and mapped (see Multimedia Appendix 1).

From the transcript and narrative structure of each personal video story, quotes about their *doing, feeling, and thinking* were extracted and initially mapped separately into 4 personal journey maps. The *stages* were framed and labeled based on the similarities of activities and interactions across the first 4 personal journey maps. We generated the commonly shared journey map and added one more personal video story (the fifth YouTuber video), extracted the quotes, and analyzed the activities and interactions to check whether we had reached theoretical saturation on the generated journey map (see Multimedia Appendix 2).

Commonly mentioned symptoms come first. We then detailed the *steps* within each stage based on the “doing” quotes in transcripts. Based on the combination of data on feelings, steps, and symptoms, the *mood experience curve* was created to clarify the generic mood experience of the patients during the whole journey, from when they became aware of incipient symptoms to quarantine and self-recovery. Since not all of these persons with mild COVID-19 went through all steps, we bolded the video timeline to indicate which steps each patient actually experienced. The *video timeline analysis* of the video duration of each stage per patient was mapped with the percentage (divided by the total video duration), indicating which stages the patient attached more importance to (see Multimedia Appendix 1). Finally, from the analysis of the *touchpoint interactions*, the specific services and products were clustered, categorized, and mapped on the resulting patient journey map (visualized in Multimedia Appendix 2).

#### Comments Thread Analysis

The YouTubers usually mentioned the purpose of publishing the video at the beginning or the end of the video, and the comments were responses to the YouTubers. From the more than 1000 comments per video, we selected those 50 comments that had interaction in the form of a follow-up comment from the YouTuber.

We thus analyzed the *video purpose* together with the comment threads to figure out the interaction between the YouTuber and the viewers, and combined these data sources to analyze the underlying purpose for sharing the home isolation experience in depth. In the transcripts, we annotated the quotes concerning why they wanted to post the video by means of initial coding, then classified the purposes mentioned by different YouTubers and synthesized them into a classification [55] of 4 themes in Multimedia Appendix 3.

Since each comment expressed more than one meaning and there were overlaps between different comments, we used an Excel (Microsoft Corporation) table to code the *comment threads*. First, we put the top 50 comments on each video in the first column in an Excel table, put the initial codes in the first row, and marked the cells where they intersected. Second, we counted how many viewers mentioned each code. Finally, we categorized the codes into 13 themes (see Multimedia Appendix 3) and pointed out how many people mentioned each theme in the 250 comments.

#### Touchpoint Needs Analysis in Relation to AI in eHealth Services

Based on this data analysis of the patient journey map and interaction between YouTubers and viewers, we synthesized key insights by inductive reasoning [56] and clustered key insights into 13 categories, leading to three identified needs of persons throughout the journey of home isolation (see Multimedia Appendix 4).

## Results

### Key Findings

The patient journey in Multimedia Appendix 2 maps the commonly shared home isolation experiences of persons with mild COVID-19 symptoms. The first key findings concerned an extensive awareness period before testing positive, experiences of less typical and more personal symptoms, a severe negative mood experience curve, and inadequate home health care service support for patients with mild COVID-19 through all stages. Second, the key finding from the analysis of the video’s purpose and the comment threads concerned the benefits and drawbacks of social media support for patients with mild COVID-19. With the third and final key findings, main touchpoint needs during home isolation were synthesized to provide opportunities for AI eHealth concepts.

### Awareness Period Before Testing Positive

The stories on personal experiences revealed a considerable period during which the YouTubers became aware of the outbreak of the virus and its public health impact before they related their symptoms to COVID-19. Although most of these persons (P2, P3, and P4) became highly aware of the public health threat (after the prestage of unconsciousness) in less than a week, some of them had low awareness (P1 and P5) and took much longer to do so—from 2 weeks to as long as 2 and a half months (stage 1). All of them went through a period of up to 4 days during which they related the public health situation to their personal condition and symptoms (stage 2), followed by 1-2 days for the testing stage (stage 3). The home isolation period (stage 4) lasted at least 1 and a half weeks but was around 1 month for most (P1 and P2). As none of the YouTubers had yet fully recovered at the conclusion of their video stories, the self-recovery period is expected to last even longer.

### Less Typical and More Personal Symptoms

Based on the overall analysis of similarities and differences, the symptoms reported in the personal stories appeared to be different from one another. Each of the patients appeared to experience their own specific symptoms. All in all, almost 50 different symptoms were reported, ranging from a mild headache, loss of smell, a stomachache, high temperature, and dizziness to the more critical symptoms of fainting, shortness of breath, and high heart rate (Multimedia Appendix 1, bottom layer). In addition, the occurrence of similar symptoms also appeared to be different over time. For instance, P1 and P2 only had a fever in stage 2, while P4 had a continuous fever until the end. In contrast to these individually experienced physical symptoms, a general consensus was found on negative feelings and deteriorating mood experiences.

### Negative Mood Experience Curve

The consensus on the negative feelings that all the YouTubers experienced concerned severe anxiety about dying and related feelings of depression and despair.

I knew I was gonna get sick and we'd go through the process that we're seeing on TV, go to the hospital, have complications and die. This is horrible to think about. It's so, so scary

P2

From the moment that they experienced their first symptom, they experienced severe negative moods that became dramatically worse when the symptoms continued to deteriorate, reaching the lowest level just before testing positive. (The mood experience curve is diagrammed on the top layer of the patient journey in Multimedia Appendix 2.) Surprisingly, testing positive was commonly experienced as an emotional relief. After this, their overall negative mood improved slowly but gradually during the home isolation period of self-recovery. That said, some of them experienced another period in which they felt emotionally broken again and then improved afterward. It is worth noting that, although not all of the patients went through the same ups and downs, overall, they all faced severe feelings of depression and mood fluctuations throughout the journey and especially in stages 2, 3, and 4.

The main differences were that P3 and P5 did not repeatedly look for medical help with no improvement in the second stage while P1 and P2 did. P3 was the only one who had not been tested and did not worry about having limited access to resources because her sister is a doctor and can get timely 24-hour professional help.

### Inadequate Home Health Care Service Support

As shown in Multimedia Appendix 2, the patient journey followed several distinct stages: prestage with unconscious and low awareness of the public health risk posed by the virus outbreak, experiencing suspected symptoms, relating symptoms to COVID-19, testing and confirmed positive, and quarantine and self-recovery.

#### Prestage: Unconsciousness

The patient journey starts in the stage in which the patient is still unaware of the situation (prestage of unconsciousness). This is the stage that the patients tried to recall to reconstruct how they were infected. All had been to public areas and crowded places such as supermarkets, cafés, and party locations.

I was at a party at one of the hotels – there are probably over a thousand people

P1

Unaware of the spread of the virus and the danger of becoming infected, most of them also continued to visit public places.

The thought that I could have been infecting other people is just horrific to me

P5

This situation caused particular feelings of guilt about their personal and public responsibility for having infected others before being diagnosed with COVID-19 (P3, P4, and P5).

#### First Stage: Experiencing Suspected Symptoms

At the beginning of the first stage, before experiencing any symptoms, some of the patients already became anxious about the news of the COVID-19 outbreak. When the first symptoms were appearing, the mood of most of the patients began to decline rapidly, worsening as they experienced more physical symptoms and paid more attention to media coverage of the abnormality of the hospital situation.

Before my family got sick, my anxiety about all this was pretty high

P2

Those who were highly aware of COVID-19 could quickly relate their own symptoms to COVID-19. Others mentioned they had little knowledge about COVID-19 until the moment when they got tested and diagnosed positive.

I'd been sick for two months and I still did not have an answer, I still had all the symptoms

P1

This had major consequences, as they had not taken enough appropriate protective measures and infected several others—the longest period a person went without diagnosis was 2 and a half months.

#### Second Stage: Relating Symptoms to COVID-19

In the second stage, when the YouTubers started to realize that they had a high possibility of being infected, some could accept it, while others could not.

It's not corona, I think it's laryngitis, fingers crossed

P3

Most became highly anxious and even panicked due to the overwhelming media coverage and “death statistics” on patients with severe COVID-19 in hospitals. They started to have dark thoughts and different levels of stress up to severe depression.

Since I got sick who would a guessed, wrote down a few notes cuz my mind is like scrambled

P2

These persons indicated that they paid too much attention to COVID-19, and the overwhelming negative information led them to live in constant anxiety. In addition, all of these persons experienced a lack of medical help and guidance. Due to this lack of help, some chose to endure all the symptoms to save medical resources for others.

I didn’t necessarily want to go to the emergency room because I didn't want to take resources away from people who needed it

P4

The only positive spark that provided a little comfort was the help they received from their family and friends.

Then all of a sudden I just fainted so I got up and I tried to go get my roommate in case anything happened

P4

Overall, due to the inadequate and ineffective support from professional health care, most of these people constantly worried about COVID-19 and its terrible consequences. All reached the lowest level of severely negative mood at the end of this stage.

#### Third Stage: Testing and Confirmed Positive

In the third stage, when the persons began to seek clinical support to test their suspicion that they had the COVID-19 virus themselves, most experienced an improvement in their mood. However, some of the others with mild symptoms were not diagnosed with COVID-19 at the first consultation due to a lack of clinical knowledge about mild COVID-19.

I did all the tests and he could not figure it out, now the one thing he did know was I was still having night sweats

P1

These persons became severely upset about the ineffective treatment they personally experienced, and their videos provided examples of incorrect diagnoses and repeated visits to health professionals.

He did a bunch of tests and they all came back negative. They didn't test me for COVID-19 though because they just said that they had to keep that for people who really needed it

P4

#### Fourth Stage: Quarantine and Self-recovering

In the fourth stage of home isolation and self-healing, the mood of all the patients tended to fluctuate several times, as they refrained from social interaction for a long time and had unstable health conditions. They still felt depressed when their health deteriorated again during isolation.

It's definitely the sickest I've ever been in my adult life

P5

They required professional guidance on the proper measures to take while in quarantine at home because they were concerned about infecting family members.

We talked to the doctors and they said like there was no reason that I had to stay separated from everybody (whole family infected)

P2

The main reasons for an improvement in mood were that the symptoms had been mild and were getting better, and the patients were taking on more activities and gradually returning to a normal life. The reasons for feeling more negative were the abnormality of life in home isolation, new severe symptoms, and ineffective treatment. Overall, the mood of these patients improved particularly after they received social support and effective treatment.

### Benefits and Drawbacks of Social Media Support

The findings from the analysis of the YouTubers’ purposes for sharing their videos and the comments made by viewers confirmed the benefits and drawbacks of the social sharing of public health experiences. The video purpose analysis revealed the commonly shared purpose of going through a difficult time together and receiving support from the audience. Reasons for sharing their personal story were to encourage viewers to pay more attention to protective measures and take social distancing seriously in public; to help viewers relieve their excessive anxiety and fear, and gain a better understanding of mild COVID-19; and to help others with similar experiences by sharing their real condition and self-recovery advice.

The findings from the comment analysis showed that the majority of the 250 commenters (n=166, 66.4%) expressed their likes and thanks to the YouTubers for sharing real COVID-19 experiences and encouraged and blessed them. Of the commenters, 25.2% (n=63) also shared their experiences and feelings, indicating that they can relate to the YouTubers. After watching the video, 13 of them said that they actually realized that they might have mild COVID-19 too. Some (n=26, 10.4%) indicated that they became scared and depressed. A minority (n=48, 19.2%) talked about the public health response to COVID-19 from governments, media, and the public, and asked people to take it more seriously. Home remedies such as vitamin C, elderberry syrup, lemons, and honey were suggested by 7.2% (n=18) of the commenters. Only 5 health care professionals commented. Inappropriate behavior such as going for a walk before having fully recovered were pointed out by 9.2% (n=23) of the commenters, and some (n=10, 4%) of the commenters made jokes.

In summary, the positive influence of personal video stories is that they reach people who are not familiar with the disease yet, they encourage viewers to take mild COVID-19 more seriously, and they provide some emotional relief; their negative influence is that they can spread disinformation and panic.

### Main Touchpoint Needs During Home Isolation

Based on these key insights of the patient journey map and interaction between YouTubers and viewers, three needs were identified.

#### Touchpoint Need 1: Stress Release

Concerning the touchpoint interactions, the patients commonly mentioned the difficulty of obtaining trustworthy information. Although information about COVID-19 was easily available from various sources such as TV, friends, and websites, the quality of these sources varies.

I think someone sent me yesterday an article with no one delaying conditions dying but it's still kind of like really freaks you out when you're home and can’t breathe

P3

The patients found it hard to judge the truthfulness of news. An incorrect perception of the disease resulted in continued aggravation of the symptoms and brought a strong sense of uneasiness and anxiety to the patients with COVID-19.

When symptoms first appeared, the YouTubers wanted to find out the cause of their physical discomfort. Due to their lack of knowledge of all the COVID-19 symptoms at the beginning of the outbreak, many of them behaved as they would with a normal disease. However, their continuous uncertainty, ineffective treatment, and deteriorating condition caused them fear and anxiety. Care professionals working in regular health care services were not able to diagnose patients with mild COVID-19 with atypical symptoms, which led to a long period of uncertainty (the longest of which was 2.5 months).

#### Touchpoint Need 2: Personal Health

Patients with mild COVID-19 had a need for professional medical guidance throughout the journey, beginning from when the symptoms appeared, with a focus on different needs at different stages.

Everyone's kind of dealing with like some symptoms but there's no confirmation because they couldn't give us the testing, so this is kind of where we are at until this next super weird symptom hit

P2

The strong feelings of uncertainty and stress due to negative thoughts caused an urgent need for testing; rejection could increase the negative impact on mental health. After testing positive, some of the patients became excited when their physical condition temporarily improved and then experienced mental breakdowns when their condition became worse again.

I just isolated at home and [did] not go out at all until three days after all the symptoms disappear[ed]

P2

It is hard for people to judge the point of recovery without a professional diagnosis.

#### Touchpoint Need 3: Social Support

In all their personal video stories, the YouTubers mentioned that their families, friends, and viewers provided them with plenty of help, ranging from basic support for living to emotional support for coping with anxiety. When they first felt a strong sense of insecurity due to the onset of weird symptoms, they longed for help from their families and friends to obtain basic necessities like food and medicine.

I find it hard to do little things like clean my teeth then go for a shower. I couldn't get my hands on any paracetamol for weeks. I really don't know what I would have done without it

P3

From the moment they suspected they were infected and went into home isolation, their internet communication became more important.

I'm separated from my family, I can't see my son or my wife

P5

I am on the phone with my friend and Facetime regularly

P3

Most of them could no longer work, study, or engage in their usual hobbies, and they experienced feelings of boredom and frustration about this, although some started to enjoy a new hobby. Overall, all of these people felt lonely and helpless during isolation. They shared their story on YouTube with the aim of helping others who had COVID-19.

## Discussion

### Principal Results

This study clarifies the stages, symptoms, mood curve, and touchpoint needs of persons with mild COVID-19 symptoms through mapping the patient journey. This design research of the systematic and in-depth analysis of how patients with mild COVID-19 told their personal stories in their self-shared videos and the comment threads found that persons with mild COVID-19 usually took an extensive period to realize that they were personally experiencing the public health threat of the virus outbreak. They all faced the same problems of severe negative and fluctuating moods while dealing with different symptoms. They lacked adequate and effective home health care service to overcome adversity. The home-isolated persons with mild COVID-19 symptoms turned to their family and friends not only for social support but also for medical assistance and obtained additional emotional support by sharing their stories on social media. Three principal touchpoint needs were identified. First, there is a need to relieve the anxiety caused by the virus by providing reliable public health information. Second, more personal health monitoring and guidance is needed to address personal symptoms. Third, more mental health guidance and social support is required to positively influence the severe moods and emotional problems of those with mild COVID-19.

The theoretical implications concern a new contribution to better understand underserved persons with mild COVID-19 symptoms during their home isolation. As a contribution to the field of AI in eHealth, we propose taking the user-centered findings and embedding them in AI eHealth service touchpoints to improve the home isolation experience.

### Proposed Service Touchpoints of AI

As the number of patients with COVID-19 is still increasing and some countries are still conducting limited testing, the shortage of global medical resources will persist in the near future, and the demand for more and better eHealth services for patients will continue to rise. To meet the urgent need for public health, it is time to put AI technologies into practice. Although persons who are unfamiliar with new technologies may be less willing to use them, research also shows that, as long as they feel that a specific eHealth service has the ability to improve the quality of treatment, they will intend to try it. Therefore, the target users for eHealth innovation with AI are those who have urgent needs for better medical service—in this study, the target was home-isolated patients with mild COVID-19 symptoms in the context of limited medical resources. In addition, as most of those with mild COVID-19 symptoms are young people, the application of AI can be easier to promote because they are generally more receptive to new technologies [50].

We translated the patient journey insights into value creation for AI innovations in eHealth [43] and designed 3 initial service concepts for an AI application. We used the insights to improve a patient’s experience with eHealth services. The three concepts are based on the premise of an eHealth app used on smart mobile devices.

#### Trustworthy Public Health Information

In this concept, persons can get more trustworthy information about COVID-19.

- *Group identification*: Identify the group of people who do not pay attention to the outbreak through social media and automatically show more information about COVID-19 in the areas of interest they often follow.
- *Dangerous area identification*: Identify dangerous areas by tracking patients who were diagnosed and public transportation data. Evaluate the hazard level. Release information about dangerous areas to remind people that they should visit these areas less often and take protective measures while paying more attention to their physical condition if they have been in dangerous areas.
- *Symptom analysis*: Collect all the atypical symptoms related to COVID-19 that are shared on the internet, reminding the public to pay attention to these symptoms. Meanwhile, facilitate the work of health professionals to better study COVID-19.
- *Information analysis*: Based on the user’s search history, provide more information on the issues that cause the most anxiety to the user and that they have the most questions about. Meanwhile, have experts identify and refute false information or rumors.
- *Positive relaxation*: Provide personalized information for users who allow the use of their data. Show more related information in line with their interests. If the user is experiencing depression because of COVID-19, present more positive information and stories of patients with mild symptoms who have recovered.

#### Personal COVID-19 Health Monitoring

In this concept, by inputting symptoms and physical condition data through text or voice, users can self-diagnose whether they have been infected by COVID-19 and follow up with self-monitoring and personalized care as well as daily predictions about their potential health condition.

- *Self-check*: AI carries out a preliminary diagnosis based on the symptoms indicated by the user and answers to questions, and provides a diagnosis result in the form of a list of all the possible causes with their probability as a percentage, especially the possibility of being infected with COVID-19.
- *Remote diagnosis*: According to the user’s recorded symptoms and physical data, the system can automatically match a suitable general practitioner or specialist for the user to communicate with while making it easier for the doctor to arrive at a diagnosis and give treatment recommendations.
- *Controllable testing process*: Based on the user’s health condition records, AI prioritizes users with severe symptoms (this process runs in the background to prevent users from recording false information because they want a test as soon as possible). AI recommends the most suitable hospital and shows the potential waiting time. All medical assistance provides a clear status report on progress and the estimated waiting time for the results to improve patients’ feeling of control.
- *Professional advice*: According to the diagnosis result, the system gives suggestions for the next steps. If the probability of infection is low, the system will suggest that the user should continue paying attention to their physical condition and take proper protective measures when going out. If the probability is high, the system will suggest that the user should go into quarantine and continuously observe the symptoms for a few more days. In addition, based on the user’s symptoms, the system will present similar cases to help users better understand the disease.
- *Self-monitoring and treatment*: Users can connect their monitoring device such as an oximeter to the app to automatically collect body data or manually record their body condition daily. The system judges the development of the disease daily based on the data. It also provides proper treatment according to the user’s health condition (eg, exercises that help recovery, suitable foods to eat, or things that the user needs to avoid). If the user’s condition constantly worsens, the system will automatically suggest that the user should consult an actual doctor. In the event of an emergency, the user can press the emergency button, and the system will match the user with the fastest medical assistance available. If the user’s physical condition becomes stable for a certain period of time, the system will inform the user that they have recovered and can go outside.
- *Personal recommendations*: Monitor users’ mood based on the recording of their health condition, voice diaries, and interactions with the app. Post examples of users with mild symptoms to show a high possibility of full recovery and make them feel positive. In the meantime, inform users with real cases of COVID-19 about what they might experience in the days ahead and how to prepare themselves for it. For example, their health condition may worsen or fluctuate over the next few weeks. Based on the keywords retrieved by the users and the content viewed, combined with the health condition record, post positive information when signals of anxiety appear. When the user’s condition has just improved, remind them that they still need to be careful and take it seriously.

#### Community Support

In this concept, users can socialize with those who have similar experiences online to get more social support.

- *Together with families*: With the consent of the user, share the users’ health and emotional condition wheir families in case of emergency to enhance their feeling of connection.
- *Peer and community wisdom*: Increased socialization while helping each other by encouraging users to post their experiences and feelings, answer questions, and participate in a discussion group. In addition, a specific “meme module” can be provided to give users a chance to reduce their stress by sharing jokes, expressing their plight, and fostering empathy. Moreover, inspire users to try new hobbies that are shared by others on the hobby discussion board to reduce their boredom. Additionally, health care experts can participate to validate the posts. Rank the videos separately based on feedback from experts and other patients.

Regarding the development of an eHealth application using AI technology and its adaptation to the continuously changing situation of mild COVID-19, we recommend that application developers should add new concepts based on an existing eHealth application. By making a preliminary prototype and validating it with a small group of patients with mild COVID-19, developers should quickly iterate to meet missing needs that have not been considered before. It is necessary to be flexible based on how the COVID-19 situation develops and as regulations are updated.

### Limitations and Implications for Further Research

Although the patient journey mapping is grounded on rigorous and systematic analysis of the qualitative data on the experiences of multiple persons with mild COVID-19 symptoms, this study has several limitations. In this design research, we used videos shared by people on YouTube as the main data source. The advantage of this self-shared data is that these patients have not been influenced by the researcher in advance, and the data is guaranteed to represent the patient perspective, which to a certain extent led the amount of information to exceed the researchers’ expectations. The disadvantage is that unilateral dialogue without questions from researchers also meant that much of the data was irrelevant to the research question, which required the researchers to spend more time on sampling, extracting, and managing the risk that the personal video stories would not provide in-depth answers to some of the subquestions on the patients’ experiences. In this regard, future research that includes additional face-to-face verification procedures is recommended to further enhance the robustness and reliability of the results. Concerning the sample strategy, the limitation of the current sample is that the participants are all YouTubers from the Americas and Europe, who tend to actively share and seek social support more easily than others. Further limitations relate to the racial and socioeconomic status disparities in online narratives that have been documented; in particular, stories by minorities are underrepresented on the internet, including on YouTube [47]. For future research and eHealth service design, more types of personal experience need to be considered. In addition, compared with other videos that are not shared on the social media platform, YouTube videos have comments from viewers under each video. From those comments, we could easily collect different viewers’ opinions on the video content, and we gained insights by analyzing those data. As most comments are composed of short sentences, future research could include AI technologies such as natural language processing and machine learning to efficiently analyze a larger number of comments.

### Conclusions

The design of the patient journey map and the underlying insights into the home isolation experience serve to uncover new knowledge and enhance the professional understanding of persons with mild COVID-19 symptoms. The journey mapping synthesized urgent needs for eHealth service touchpoints, for instance, that patients require reliable public health information, personalized health monitoring guidance, and social support. To overcome the inadequate service provision challenges that became apparent in mapping the journey, initial service concepts were proposed for new AI eHealth services to improve the experience of patients with COVID-19 by providing effective health care guidance.

## Appendix

Multimedia Appendix 1 Personal video story coverage and experienced symptoms during home isolation.

Multimedia Appendix 2 Patient journey map of persons with mild COVID-19 during home isolation.

Multimedia Appendix 3 Video purpose and comments coding trees.

Multimedia Appendix 4 Visual summary of design research.

## Abbreviations

AI: artificial intelligence
CEST: Central European Summer Time
WHO: World Health Organization
