# Correction: A Patient Journey Map to Improve the Home Isolation Experience of Persons With Mild COVID-19: Design Research for Service Touchpoints of Artificial Intelligence in eHealth

**DOI:** 10.2196/29794

**Published:** 2021-05-04

**Authors:** Qian He, Fei Du, Lianne W L Simonse

**Affiliations:** 1 Department of Design Organisation & Strategy Faculty of Industrial Design Engineering Delft University of Technology Delft Netherlands

In “A Patient Journey Map to Improve the Home Isolation Experience of Persons With Mild COVID-19: Design Research for Service Touchpoints of Artificial Intelligence in eHealth” (JMIR Med Inform 2021;9(4):e23238) the authors noted one error.

In the originally published manuscript, Multimedia Appendix captions incorrectly appeared as follows:

Multimedia Appendix 1: Patient journey map of persons with mild COVID-19 during home isolation.

Multimedia Appendix 2: Visual summary.

Multimedia Appendix 3: Video purpose and comments coding trees.

Multimedia Appendix 4: Personal video story coverage and experienced symptoms during home isolation.

In the corrected version of the manuscript, Multimedia Appendix captions have been corrected to:

Multimedia Appendix 1: Personal video story coverage and experienced symptoms during home isolation.

Multimedia Appendix 2: Patient journey map of persons with mild COVID-19 during home isolation.

Multimedia Appendix 3: Video purpose and comments coding trees.

Multimedia Appendix 4: Visual summary of design research.

The correction will appear in the online version of the paper on the JMIR Publications website on May 4, 2021, together with the publication of this correction notice. Because this was made after submission to PubMed, PubMed Central, and other full-text repositories, the corrected article has also been resubmitted to those repositories.

