# Supplementary figures and images for: A Patient Journey Map to Improve the Home Isolation Experience of Persons With Mild COVID-19: Design Research for Service Touchpoints of Artificial Intelligence in eHealth
Source: JMIR Med Inform. 2021 Apr 12;9(4):e23238. doi: 10.2196/23238 (PMC8043148; doi:10.2196/23238)

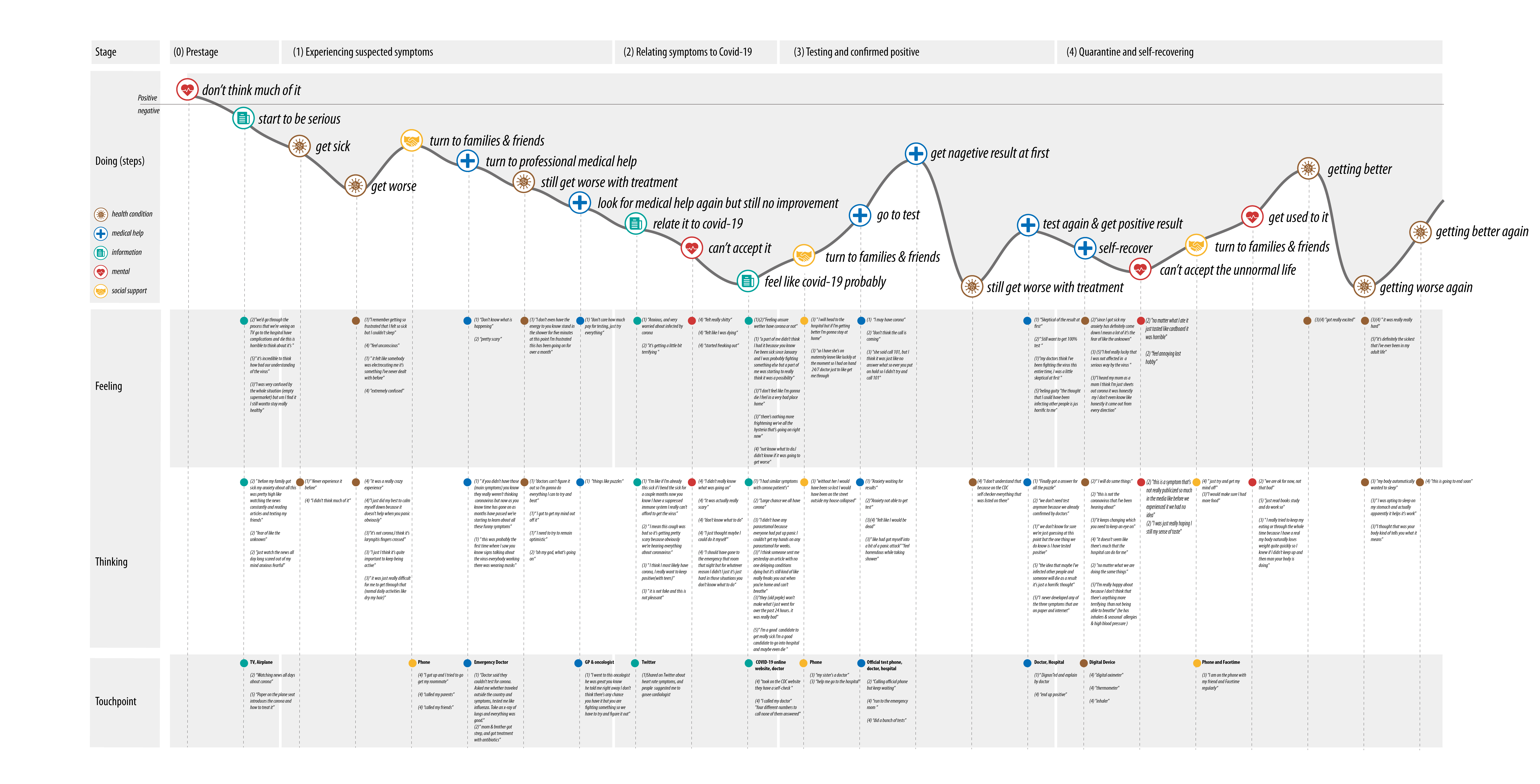

Supplement: Multimedia Appendix 1 [file medinform_v9i4e23238_app1.png]

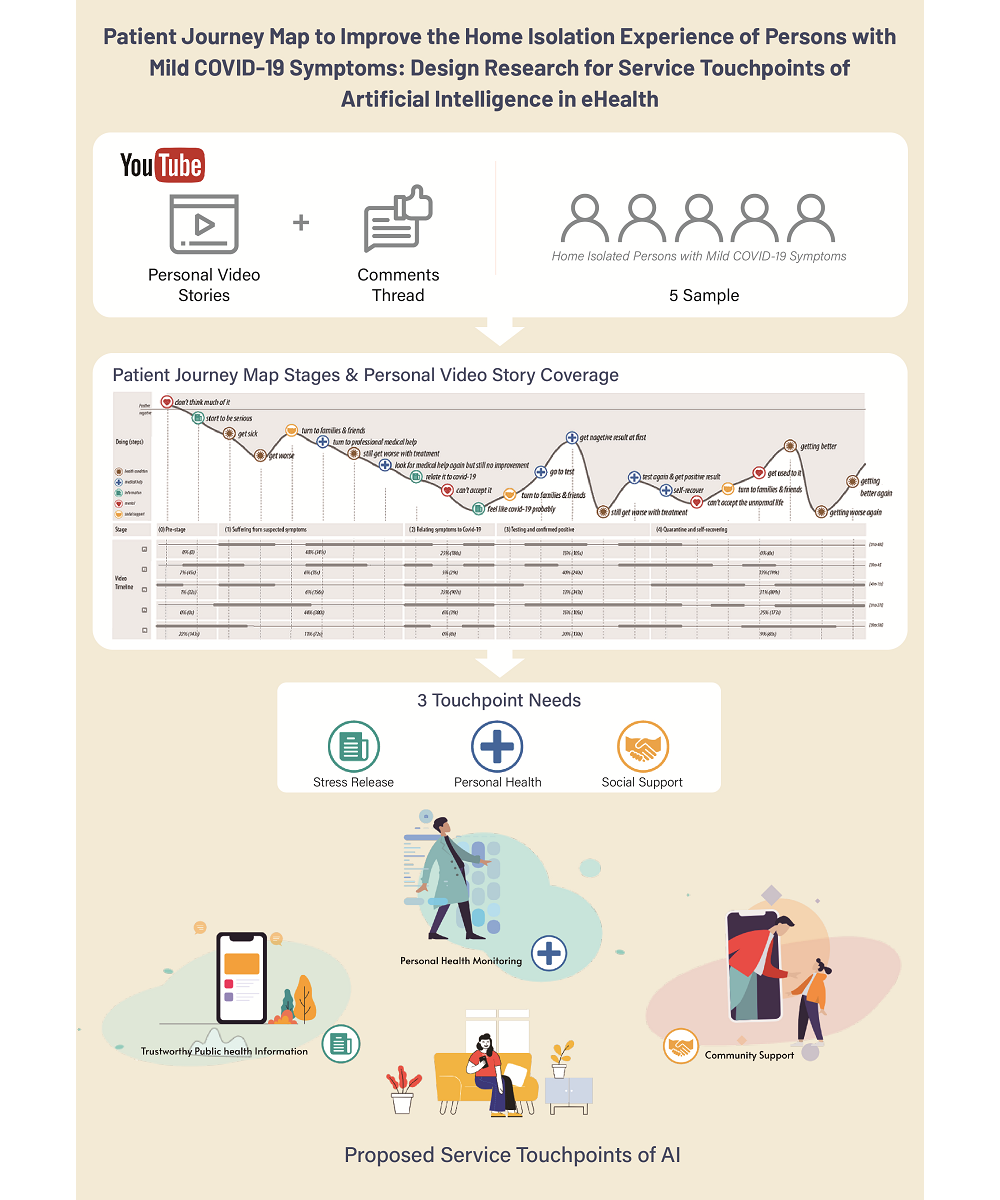

Supplement: Multimedia Appendix 2 [file medinform_v9i4e23238_app2.png]

## Appendix 1: Video Purpose and Comments Coding trees


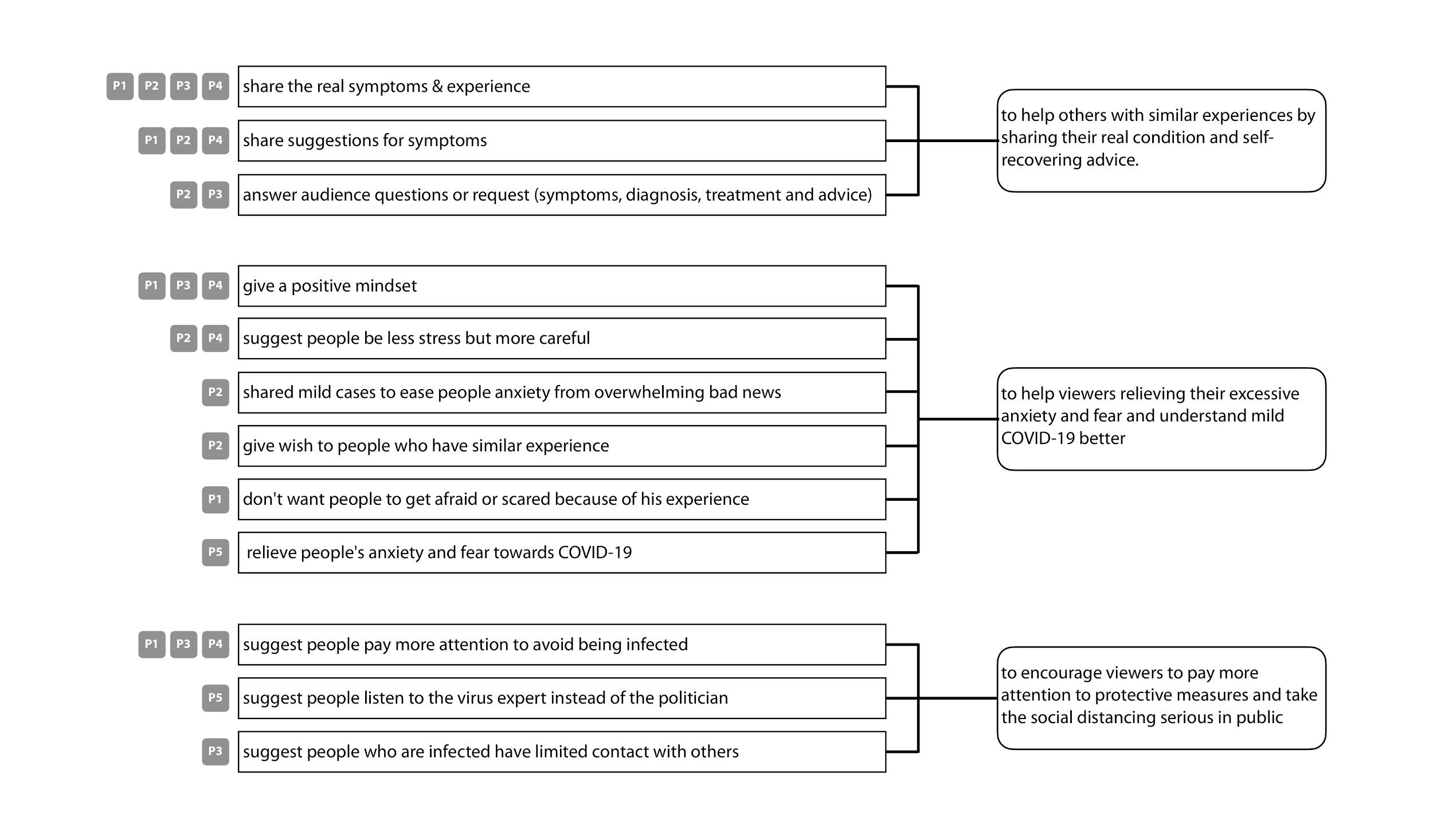


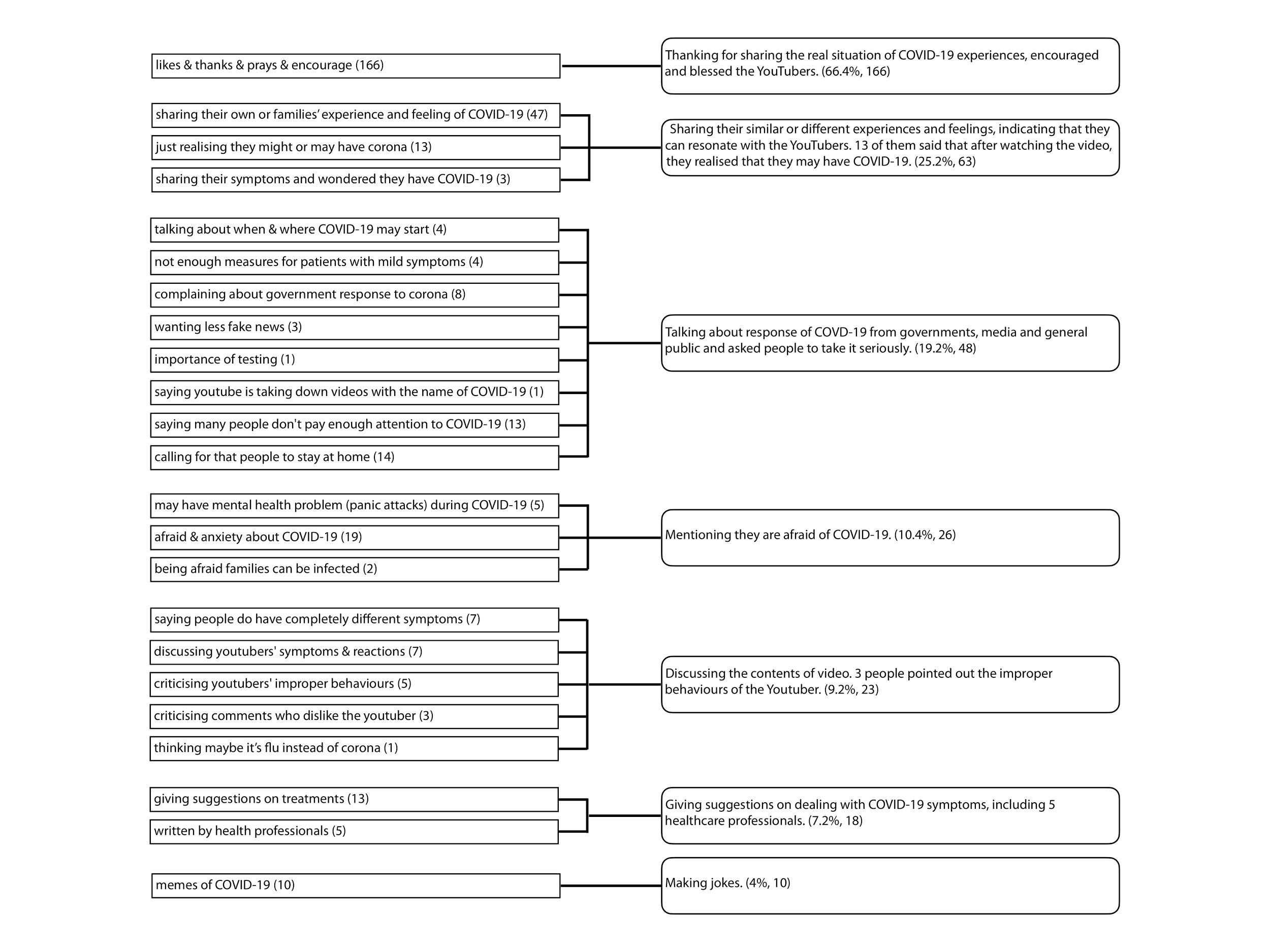

Supplement: Multimedia Appendix 3 [file medinform_v9i4e23238_app3.docx]

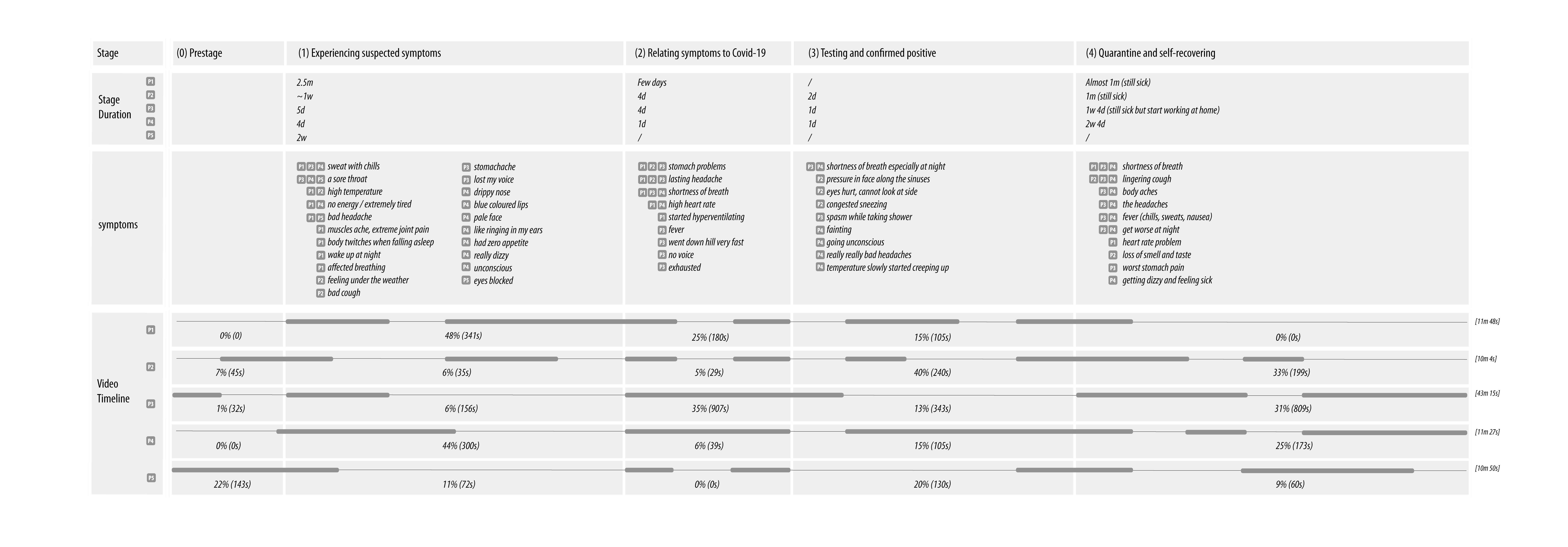

Supplement: Multimedia Appendix 4 [file medinform_v9i4e23238_app4.png]
